# Superficial temperature distribution patterns before and after physical activity in school children are indicative for personalized exercise coaching and disease prevention

**DOI:** 10.1007/s13167-021-00262-1

**Published:** 2021-11-05

**Authors:** Agnieszka Dębiec-Bąk, Anna Skrzek, Halina Podbielska, Olga Golubnitschaja, Małgorzata Stefańska

**Affiliations:** 1 Department of Physiotherapy in Motor Organ Dysfunctions, Faculty of Physiotherapy, Wroclaw University of Health and Sport Sciences, al. Paderewskiego 35, 51-612 Wrocław, Poland; 2 Department of Biomedical Engineering, Faculty of Fundamental Problems of Technology, Wrocław University of Science and Technology, Wybrzeże Wyspiańskiego 27, 50-370 Wrocław, Poland; 3 Predictive, Preventive and Personalised (3P) Medicine, Department of Radiation Oncology, University Hospital Bonn, Rheinische Friedrich-Wilhelms-Universität Bonn, 53127 Bonn, Germany

**Keywords:** Predictive preventive personalized medicine (PPPM/3PM), Thermoregulation, Thermography, Age-dependent, Body temperature, Physical activity, Patterns, Body mass, Height, Fatigue, Indicator, Personalized coaching, Multi-factorial, Thermovision analysis, Stratification, Assessment tool, School children, 7–12 years old, Fitness training, Innovative approach, Socioeconomic disparity, Healthcare policy

## Abstract

**Background:**

Thermoregulation is highly individual and predictive for potentially cascading pathologies. Altered and deficient thermoregulation is considered an important diagnostic indicator which can be of great clinical utility for specialized screening programs and individualized prediction and prevention of severe pathologies triggered early in life.

**Working hypothesis:**

Individual thermoregulation can be objectively assessed by thermovision camera before and after exercises in school children stratified by age and gender that may be of great clinical utility for personalized training early in life in the framework of 3P medicine.

**Study design:**

In this study, 60 female and male primary school children were exposed to physical exercises in the form of 45-min general fitness training. The subjects under examination were stratified by age: group 1 (7-year-olds), group 2 (9-year-olds), and group 3 (12-year-olds). Superficial body temperature patterns were measured by means of thermovision camera before and immediately after exercises, as well as after the 15-min recovery time. Temperature patterns were analyzed in 12 areas of the body front and back, covering trunk and upper and lower limbs.

**Results:**

The obtained results revealed an individual and age-depended difference in response of the body to exercises. *The first measurement prior to exercise (measurement 1)* revealed no statistically significant differences in the mean surface temperature of all analyzed areas between 7- and 9-year-old children. Further, 7- and 9-year-old children did not differ significantly in the mean temperature recorded in the trunk compared to the 12-year-old children. However, in 12-year-old children, statistically significant higher values of the mean temperature of the upper and lower limbs, were observed compared to the group of 7-year-olds and significantly higher values of the mean temperature of the lower limbs compared to the group of 9-year-olds. *Immediately after exercises (measurement 2),* a statistically significant decrease in the temperature was noted in all groups and in all areas of the body. The greatest temperature change was observed in 12-year-olds, while the least one was measured in the youngest subjects. The statistically significant relation between the average trunk temperature of 7-year-old and 12-year-old children was observed: lower values of the mean temperature of the front and back of the trunk were noted in the group of 12-year-old children compared to the group of 7-year-olds. A significantly lower average temperature of the back of the trunk compared to the youngest group was also recorded in 9-year-old children. *The study performed after the 15-min recovery time (measurement 3)* showed an increase in the average temperature of all analyzed areas. In all subjects, the mean temperature recorded in measurement 3 did not differ significantly from the initial ones (measurement 1, prior to exercises). Only the mean temperature of the trunk back of 12-year-old children was significantly lower after the rest period compared to the initial examination. In all groups, the temperatures after exercises followed by a 15-min recovery returned to the initial ones, except of the trunk backs of 12-year-old children, where the temperature was lower than before exercises.

**Conclusions and expert recommendations in the framework of 3PM:**

Thermovision analysis is an effective tool to assess individual thermoregulation and to stratify school children for personalized exercise coaching. Body exercise-based disease prevention early in life is effective when tailored to the person: multi-parametric guidance for prescribing exercises individually is needed. Contextually, proposed individualized training approach should be adapted to the age-dependent particularities and individual thermoregulation.

## Introduction

### Physical activity is highly effective for disease prevention

The physical activity is in focus of many national and global recommendations, pointing out its great potential of diseases prevention [1–4]. Regular participation in physical exercises of children and youth have many health benefits, as, e.g., enhancing muscular strength, promoting bones healthiness, and positively influencing cardio-respiratory system [5, 6]. Positive impacts towards mental health are also observed [7]. Physical activity in children should be specially promoted as it is a good prevention tool of adolescent obesity [8–10].

However, the optimal doses of health-enhancing physical activity of an individual are difficult to determine. During physical activity, numerous changes in the human body are observed. Activation of the sympathetic nervous system speeds up the transport of blood and, consequently, oxygen. Temperature increase is one of the signals sent to the brain informing about the body fatigue [11].

### Thermoregulation is indicative for health status and adaptive mechanisms’ functionality

Thermoregulation processes determine the heat balance and ensures thermal homeostasis of the living organisms. This ability develops along with other systems in the process of human ontogenesis. Maintaining thermoregulatory processes at the appropriate level depends on the degree of thermoregulatory efficiency of each person and capacity of the circulatory and respiratory systems. The production of heat in the body influences numerous metabolic processes in cells and muscle activity. Metabolism, regulated by hormones (e.g., thyroxine, testosterone, adrenaline, noradrenaline), also affects the temperature changes. A healthy human maintains almost constant inner organs temperature. The changes are small and oscillate around 0.6 °C. The situation is different for superficial skin temperature. This parameter reflects numerous factors, as inner temperature and efficacy of skin microcirculation, but also depends on the ambient temperature and physical activity. When performing strenuous exercises, human skin may reach the temperature even up to 38.3–40 °C. When the body is subjected to cold, it reduces the skin temperature to 35.5 °C or even lower. The ability to maintain a constant inner body temperature is determined by the metabolic and thermoregulatory efficiency [12, 13].

The hypothalamus is responsible for integrating incoming information from thermos-detectors located in the skin, in the spinal cord, in other parts of the brain, and from receptors in the hypothalamus itself [14].The stability of maintaining body temperature depends on the balance between the processes of heat generation and dispersion. Thermal balance is a dynamic process that requires the continuous adjustment of individual thermoregulatory mechanisms to environmental conditions [15].

### Body temperature patterns are instrumental for individualized physical activity

There are some reports on the relationship between physical performance and the tolerance of body temperature increase. McLellan’s [16] research indicates that regular physical exercises have a positive effect on the efficiency of the circulatory system, which translates into the possibility of a greater increase in internal temperature until the body feels tired. Drust et al. [17] revealed that body fatigue does not occur as a result of the accumulation of exercise metabolites, but as a result of a significant increase of internal temperature, which has an adverse effect on brain work. Improving the ability to efficiently remove heat from the body is important, e.g., to athletes as it allows them to continue strenuous exercises [18].

In children, as the entire body still develops, the thermoregulatory abilities change progressively. Biological maturation of each system occurs individually. Thermal control in children is not yet fully developed, which means that their resistance to thermal stress is much weaker compared to adults [19].

### Thermoregulation and individualized physical activity early in life: application of 3PM concepts

Children and the elderly react differently to changes in ambient temperature. Their thermoregulatory processes cope with excessive increase or decrease of ambient temperature in a less effective way. Noteworthy, newborns have undeveloped resistance against cold provocation and therefore demonstrate deficient thermoregulation [20]. Therefore, analyzing age-stratified thermoregulation in children in response to individualized physical activity is a very important issue. Children employ different thermoregulatory strategies than adults [21]. Regarding heat-dissipation, child body engages more dry heat exchange, while in adults, it is evaporative heat loss; thus, children have limited ability to lose heat through evaporation. Promoting physical activity early in life is crucial for maintaining physically and mentally vibrant throughout the life. Therefore, research activities dedicated to functioning children’s thermoregulatory processes in response to physical effort are of great importance. To this end, thermographic approach is instrumental in the area. The analysis of surface body temperature through thermography provides useful information on the efficiency of thermoregulatory processes [22]. Infrared thermography, as a non-invasive non-contact method, found some applications in contemporary medicine. It can be used for detection of different diseases like, i.e., tumors [23]. Thermal temperature mapping has also been applied for skin lesion differentiation [24–26]. The distribution of superficial temperature is a significant parameter in the assessment of various medical physiotherapeutic procedures, as well as in sports medicine [27–30].

## Working hypothesis

Individual thermoregulation can be objectively assessed by thermovision camera before and after exercises in school children stratified by age and gender that may be of great clinical utility for personalized training early in life in the framework of 3P medicine.

## Study design

### Study participants

The research group consisted of 60 pupils of both sexes of primary school. The subjects were divided into three age groups, each of 20 persons. Group 1 consisted of first-grade students at the age of 7 years, with an average body weight of 25 kg (± 3.7) and a height of 124.2 cm (± 5.2); group 2 included third-grade students at the age of 9 years, with an average body weight of 34.5 kg (± 5) and a height of 141.6 cm (± 6.5); group 3 comprised sixth-grade students, 12 years, with an average body weight of 40.3 kg (± 5.5) and a height of 148.4 cm (± 6.4). The inclusion criteria were age matching one of these three groups, good general health of the child, no injuries that would prevent from training, and consent of a parent or legal guardian. The exclusion criteria included poor general health of the pupil and doctor’s recommendation excluding the child from the active participation in physical education classes. The group characteristics are depicted in Table 1.

**Table 1:** Body mass and height in the examined groups

Group 1	Group 2	Group 3
Groups characteristics; body mass and height		
25 kg (± 3.6)	34.5 kg (± 5)	40.3 kg (± 5.5)
124.2 cm (± 5.2)	141.6 cm (± 6.5)	148.4 cm (± 6.4)
Girls characteristics; body mass and height		
24.6 kg (± 3.9)	34.1 kg (± 4.8)	39.0 kg (± 5.2)
122.8 cm (± 5.0)	140.8 cm (± 7)	150.4 cm (± 5.0)
Boys characteristics; body mass and height		
25.4 kg (± 3.4)	34.9 kg (± 5)	41.6 kg (± 5.3)
125.6 cm (± 4.8)	142.5 cm (± 5.7)	146.4 cm (± 6.9)

### Methods

The general physical exercises for primarily school kids are organized routinely 3 times per week, and lessons’ duration is 45 min. The research project included three thermographic measurements of the superficial body temperature of the front and back of the body related to one 45-min long lesson. The thermal images were recorded in identical research conditions, in a research room with an ambient temperature of 21–22 °C and relative humidity of 34–35%. During the thermal recording in research room, only one child and one researcher with thermal camera were present. The research protocol was the same for each group.

Measurement 1 was performed before general physical exercises, and measurement 2 was taken immediately after the exercises while measurement 3 after 15 min of recovery time period. For each measurement, the thermal images were captured by means of ThermoVision FLIR SYSTEM T335 infrared camera (320 × 240 pixel resolution, a 50mK NETD/0.05 °C thermal sensitivity and an extended temperature measurement range of -20 °C to 650 °C).

**Fig. 1:**
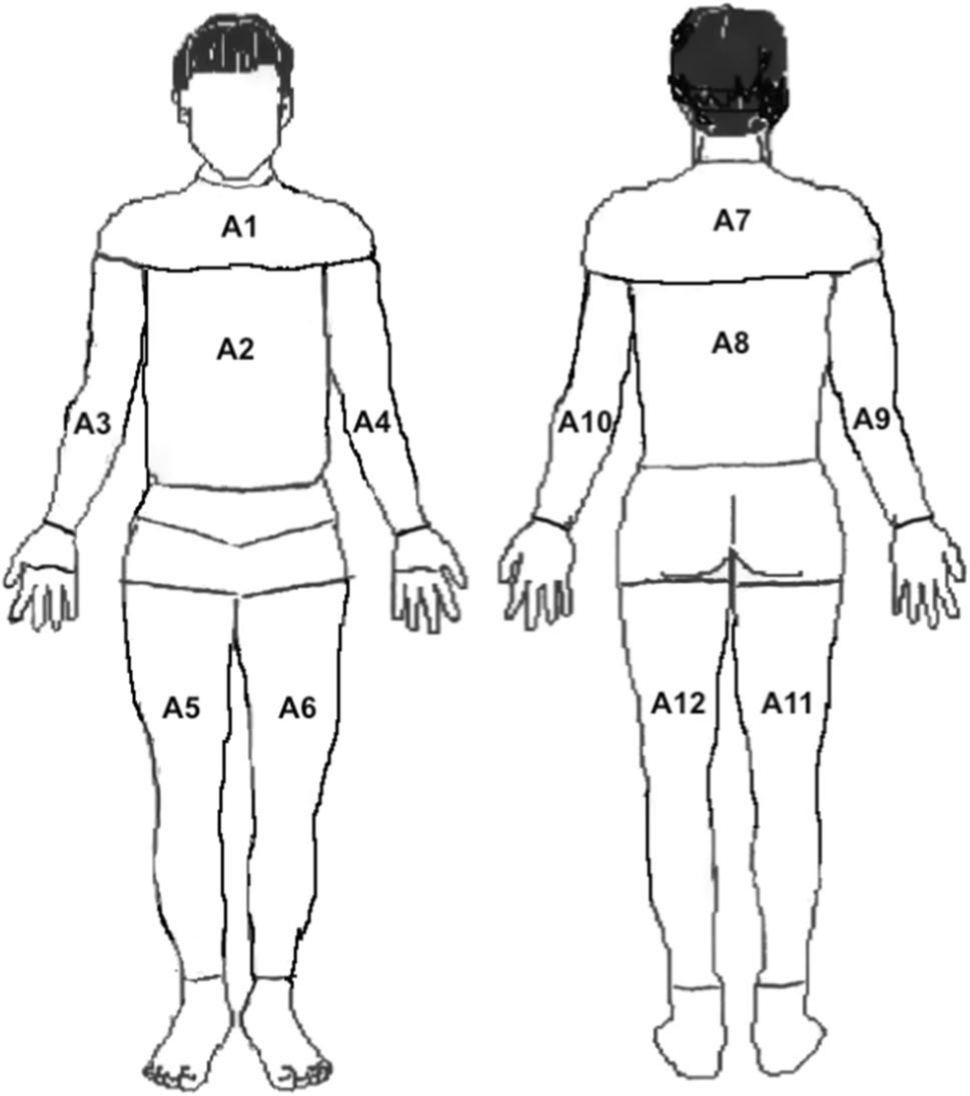
Schema presenting the parts of the body in which the average superficial temperature recorded by the thermographic camera was analyzed

The average temperature was analyzed in the front and back of trunk and upper and lower limbs, according to the schema presented in Fig. 1. The ThermaCAM Researcher Pro 2.10 software developed by FLIR Systems AB was exploited for image analysis. The average temperature changes in the following 12 body regions were determined: trunk area: front part (A1, A2), back part (A7, A8), upper limbs (A3, A4, A9, A10), lower limbs (A5, A6, A11, A12) (Fig. 1).

The training unit performed by the subjects was a modification of the seven fundamental movement patterns of the FMS (the functional movement screen) [31, 32]. The exercises were preceded by a warm-up consisting of marching on the spot, trotting, and exercises activating the shoulder and hip joints in all planes of mobility. The duration of the initial part of the exercise was 10 min. Next, the main part of the training with progressive effort was used, which consisted of a modification of the seven fundamental movement patterns tests of the FMS. Its duration was 30 min. Each movement pattern was repeated ten times. The following elements were included:

- Shoulder girdle exercises in all planes and with a load (0.5–1 kg) above the head (overhead squats): they improve central stability and stability of shoulder joints and upper limbs strength.
- Dumbbell overhead squats, squat jumps, squats with a cane in front or on the shoulders: they activate the gluteal muscles, biceps, quadriceps femoris, and muscles of the trunk.
- Single leg squats: they develop leg strength and have a strong stabilizing effect on the ankle and knee.
- Split squats: they increase flexibility, balance, and strength of the leg.
- Stabilization exercises on a stable and unstable ground (Bobath balls and mats):
- Supported kneel with uplifts of opposing limbs, e.g., upper right limb–bottom left limb
- Front holds on the forearms, i.e., the so-called forearm plank
- Front and back holds with the rise of one of the lower limbs
- Side plank with the rise of one of the lower limbs

The following equipment was used to carry out the training unit: TheraBand tape with low resistance level, exercise canes, medicine and gym balls, balance soft disks, dumbbells, and exercise benches.

The part of the training unit in the form of recovery time after physical activity consisted of a slow march, reducing the movement amplitude until stop, and breathing exercises in a standing position. Its duration was 5–7 min.

The temperature changes determined from thermographic images were analyzed by statistical methods using Statistica software version 13.l from StatSoft with a license for the University of Physical Education in Wrocław. The Shapiro–Wilk test was used to check the normality of the distribution of the analyzed parameters. Statistical variables were described using the arithmetic mean and standard deviation. The assessment of differences between the analyzed variables for girls and boys was based on the results of multivariate analysis of variance. In the case of obtaining statistically significant mean differences between the compared groups, Scheffe’s post-hoc test was used. The significance of the mean differences between the results obtained for boys and girls was checked by the Student’s *t*-test for independent samples. In all used statistical analyses, test, and coefficients, values at the level of p < 0.05 were considered statistically significant.

The research was approved by the Senate Committee of Ethics of Scientific Research at the University School of Physical Education (Permission 28.06.2007). The research participants and their parents or guardians were informed about the purpose and course of the study. Informed and voluntary consent was obtained for conducting the thermos-vision recordings.

## Results

**Fig. 2:**
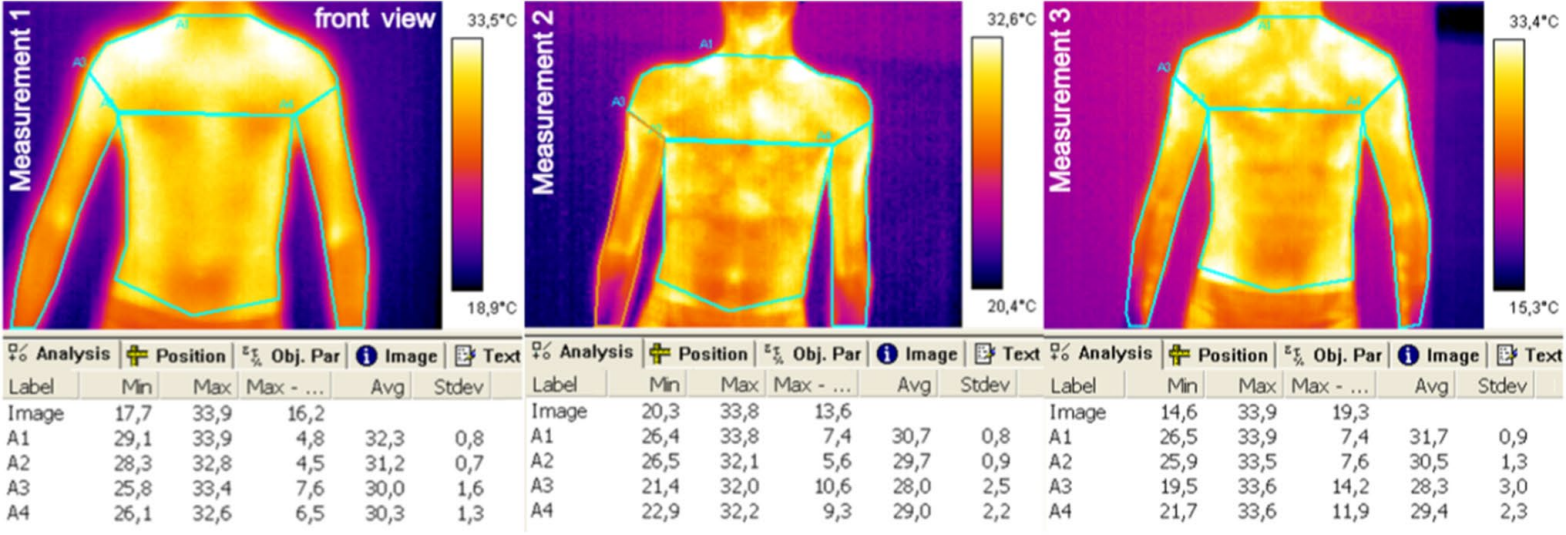
Exemplary thermal images of the trunk and upper limbs, front view

**Fig. 3:**
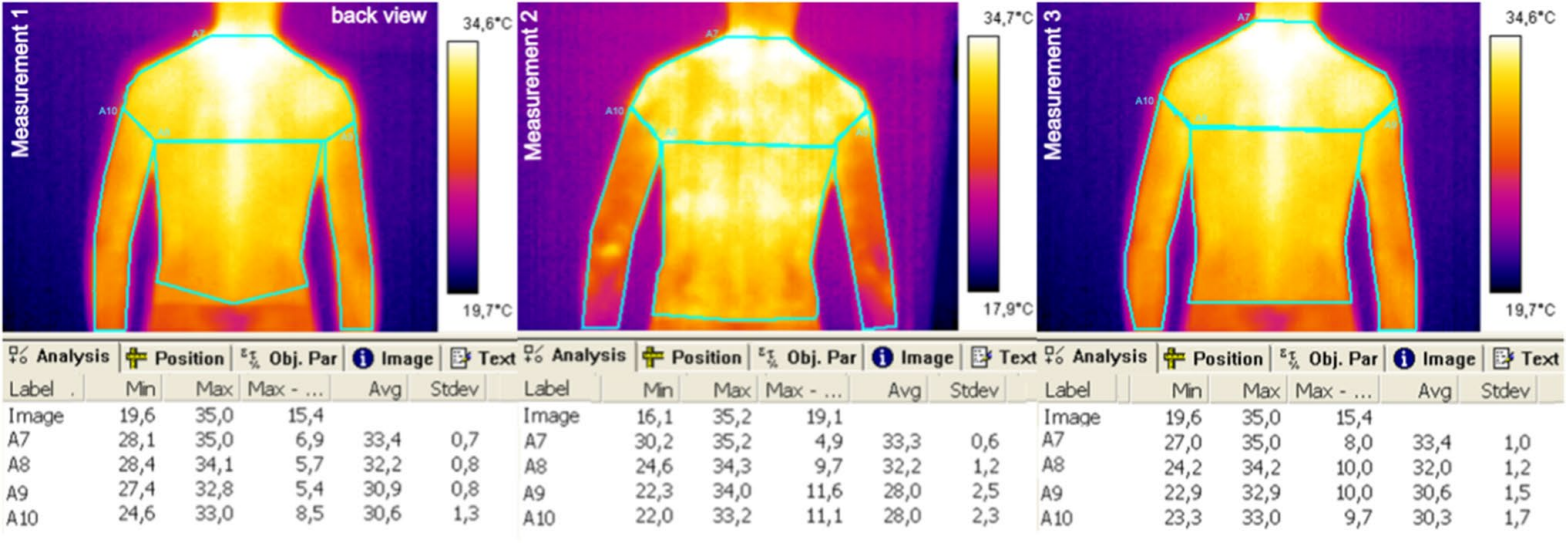
Exemplary thermal images of the trunk and upper limbs, back view

**Fig. 4:**
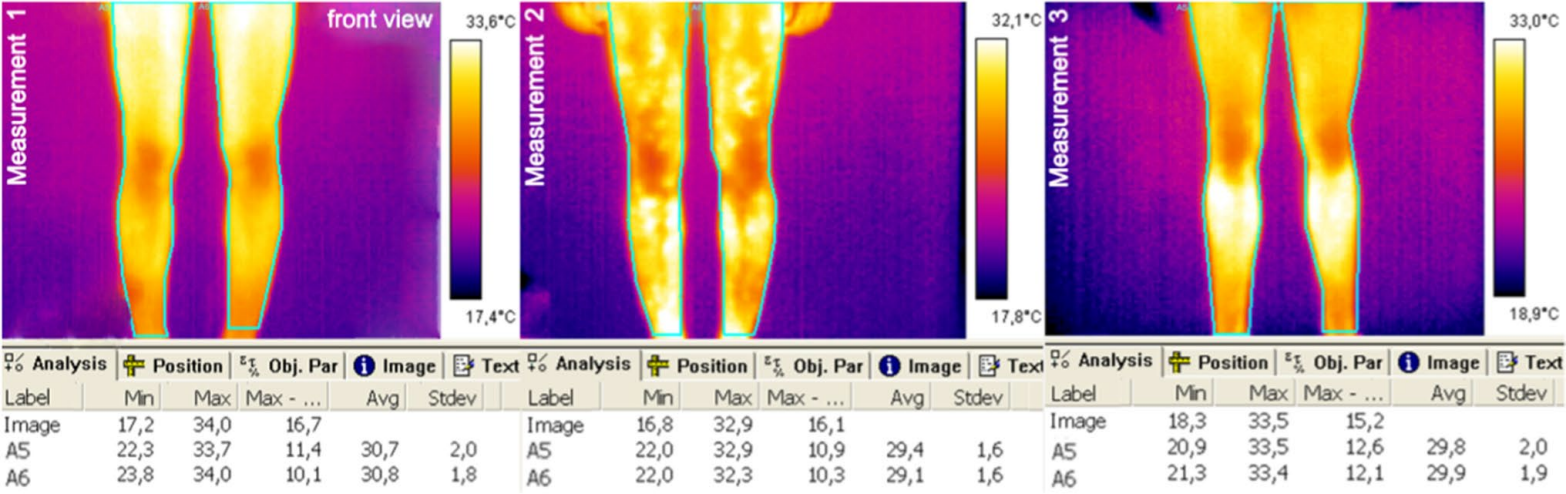
Exemplary thermal images of the lower limbs, front view

**Fig. 5:**
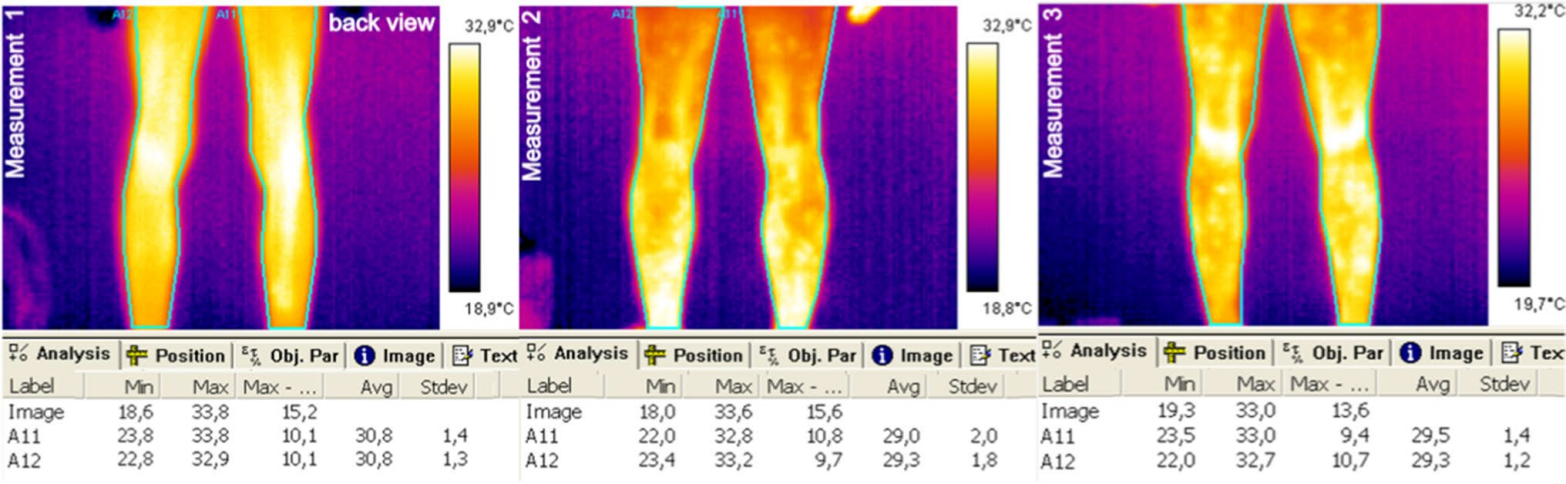
Exemplary thermal images of the trunk and upper limbs, back view

For each person, for each body area, the temperature distribution was recorded three times, as it was described above. Figures 2, 3, 4, and 5 represent exemplary recorded thermal images of analyzed body areas before general physical exercises (measurement 1), immediately after the exercises (measurement 2), and then after 15 min of recovery time (measurement 3).

The performed Student’s *t*-test for repeated measurements for both sexes did not show differences at the level of statistical significance (Table 2). Therefore, in further analyses, only age differentiations were considered.

**Table 2:** Age-depended temperature differences in boys and girls, results of Student’s *t*-test

Body part	Differences between girls and boys		
*p*-value	Group 1	Group 2	Group 3
Measurement 1—before exercises			
Trunk-front	0,6830	0,6444	0,6762
Trunk-back	0,5351	0,1656	0,8885
Upper limbs	0,8689	0,5474	0,2452
Lower limbs	0,4974	0,1055	0,1021
Measurement 2—after exercises			
Trunk-front	0,2281	0,5516	0,4914
Trunk-back	0,1128	0,0223*	0,8776
Upper limbs	0,2750	0,0996	0,5595
Lower limbs	0,1007	0,0963	0,2506
Measurement 3—after recovery time			
Trunk-front	0,2489	1,0000	0,0423*
Trunk-back	0,4403	0,1419	0,6870
Upper limbs	0,0198*	0,7354	0,5337
Lower limbs	0,3543	0,4301	0,8031

^*^Statistically significant value (*p* < 0.05)

**Fig. 6:**
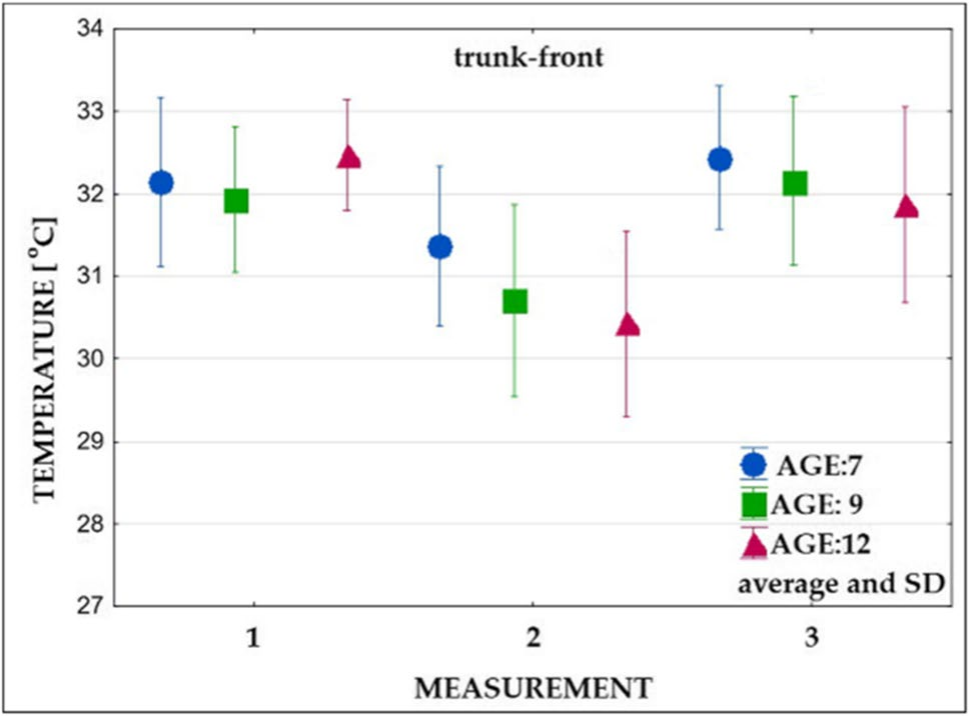
Changes in the superficial temperature of the trunk’s front part in three age groups (measurements: 1, 2, and 3)

**Fig. 7:**
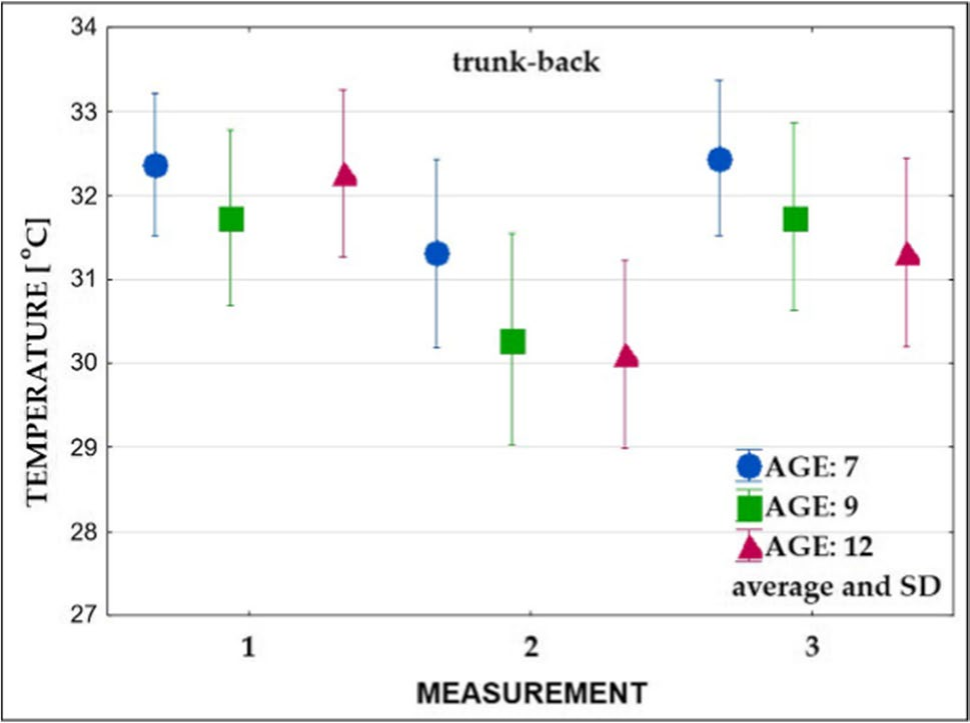
Changes in the surface temperature of the trunk’s back part in three age groups (measurements: 1, 2, and 3)

**Fig. 8:**
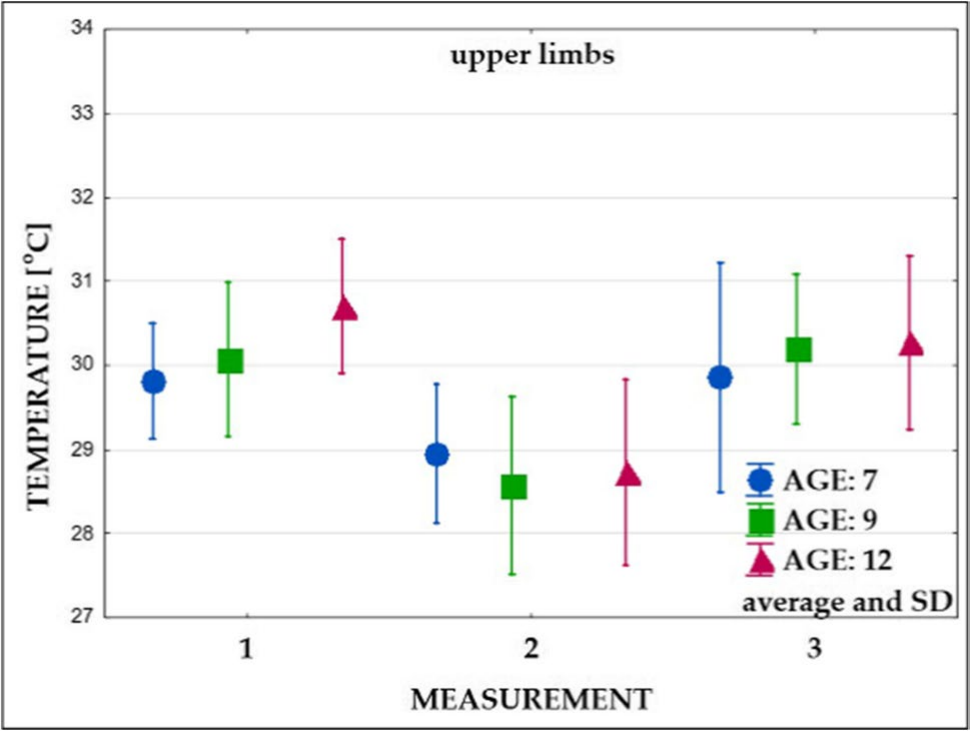
Changes in the superficial temperature of the upper limbs in three age groups (measurements: 1, 2, and 3)

**Fig. 9:**
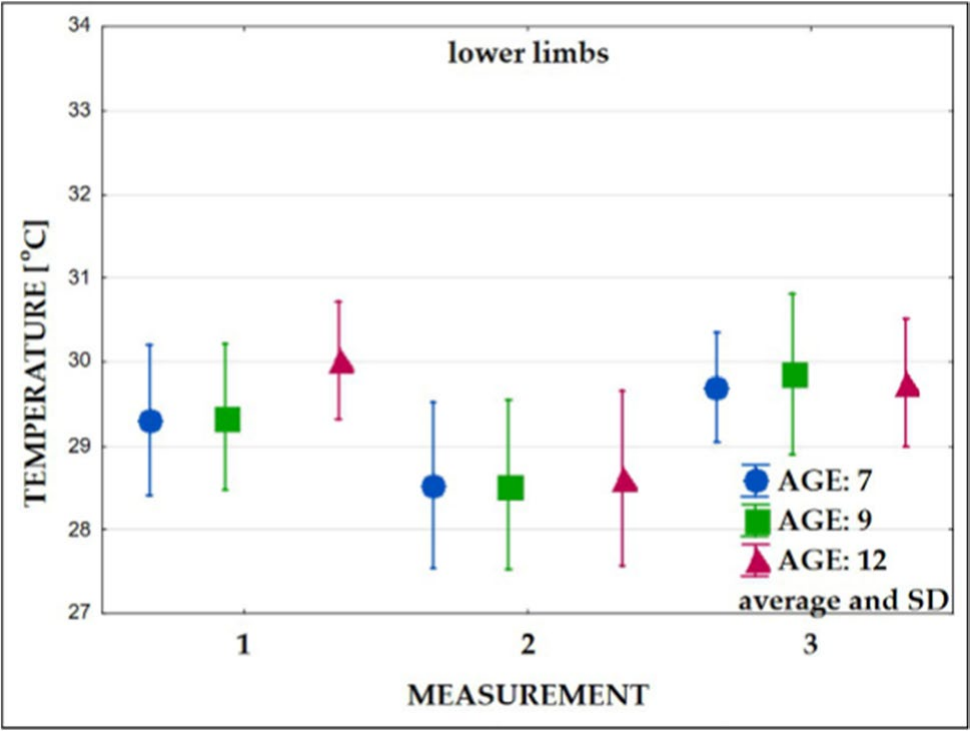
Changes in the surface temperature of the upper limbs in three age groups (measurements: 1, 2, and 3)

In the next step, the average temperatures and standard deviations were calculated for examined body areas for measurements 1, 2, and 3 (Figs. 6, 7, 8, and 9).

### Mean surface temperature prior to exercises

Measurements carried out before the exercises (Table 3, measurement 1) showed no significant differences in the mean surface temperature of all analyzed areas between 7- and 9-year-old children. Further, 7- and 9-year-old children did not differ significantly in the mean temperature recorded in the trunk compared to the 12-year-old ones. On the other hand, in 12-year-old children, statistically significant higher values of the mean temperature of the upper and lower limbs were observed compared to the group of 7-year-olds and significantly higher values of the mean temperature of the lower limbs compared to the group of 9-year-olds.

**Table 3:** Significance of differences in surface temperature values between the groups of comparison (measurements: 1, 2, and 3)

	Group 1 vs group 2	Group 1 vs group 3	Group 2 vs group 3
Measurement 1—before exercises			
Trunk-front	0.7450	0.5100	0.1627
Trunk-back	0.1285	0.9377	0.2426
Upper limbs	0.6062	0.0041*	0.0544
Lower limbs	0.9890	0.0312*	0.0442*
Measurement 2—after exercises			
Trunk-front	0.1689	0.0295*	0.7140
Trunk-back	0.0281*	0.0078*	0.8856
Upper limbs	0.4969	0.7806	0.8894
Lower limbs	0.9999	0.9644	0.9674
Measurement 3—after recovery time			
Trunk-front	0.6869	0.2349	0.6959
Trunk-back	0.1249	0.0060*	0.4513
Upper limbs	0.6308	0.4981	0.9752
Lower limbs	0.8177	0.9764	0.9168

^*^Statistically significant value (*p* < 0.05)

### Mean surface temperature immediately after exercises

Immediately after exercise (Table 3, measurement 2), a temperature decrease in all groups was observed (Figs. 6, 7, 8, and 9). Significantly lower values of the mean temperature of the front and back of the trunk were recorded in the group of 12-year-old children compared to the group of 7-year-olds. A significantly lower average temperature of the back of the trunk compared to the youngest group was also recorded in 9-year-old children.

### Mean surface temperature after the 15-min recovery time

The study performed after the 15-min recovery time (measurement 3) showed an increase in the average temperature of all analyzed areas (Figs. 6, 7, 8, and 9). In all subjects, the mean temperature recorded in measurement 3 did not differ significantly from the initial temperature (Table 3, measurement 2). Only the mean temperature of the trunk back of 12-year-old children was significantly lower after rest compared to the initial examination (Figs. 8 and 9, Table 3).

*The obtained results revealed an individual and age-depended difference in response of the body to exercises.* Immediately after physical exertion (measurement 2), the surface temperature of the whole body decreased in all subjects. The largest drop was recorded in the group of 12-year-olds. The changes ranged from 1.3 to 2.4 °C. Compared to measurement 1, smaller differences were noted in 7-year-olds (from 0.7 to 1.2 °C).

Scheffe’s post-hoc test was conducted to perform a detailed analysis of the differentiation of the results. The regression of the examined parameters in the trunk area showed statistically significant differentiation in each age group. A statistically significant difference between measurements 1 and 2 was observed in each age group for the average trunk temperature and lower limbs and upper limbs in the group of children aged 9 and 12 (Table 4).

**Table 4:** The significance of differences in surface temperature values obtained in each age group between the subsequent measurements (measurements: 1, 2, and 3)

	Group 1	Group 2	Group 3
Trunk-front			
1vs2	0.0276*	0.0000*	0.0000*
1vs3	0.9576	0.9932	0.2415
Trunk-back			
1vs2	0.0006*	0.0000*	0.0000*
1vs3	0.9999	1.0000	0.0049*
Upper limbs			
1vs2	0.1518	0.0001*	**0.0000**
1vs3	1.0000	1.0000	0.9316
Lower limbs			
1vs2	0.0082*	0.0043*	0.0000*
1vs3	0.7018	0.3152	0.9584

^*^Statistically significant value (*p* < 0.05)

1, 2, 3—measurements 1, 2, and 3

## Data interpretation and discussion

### Thermoregulation mechanisms in children are under development

Compared to adults, thermoregulation mechanisms in children are under development [33], so the resistance to thermal stress associated with physical activity could be lower than in adults. The efficiency of thermoregulation depends on the proper functioning of other systems, e.g., the respiratory, circulatory, and endocrinal. It should also be noted that the ability to adapt to strenuous exercise is lower in children compared to adults, which is why the energy cost of physical work performed by the child is higher.

### Thermography as an important diagnostic tool

The use of thermography as a diagnostic tool for the analysis of thermal patterns in the superficial body layers after physical activity enables to asses indirectly the thermoregulatory mechanisms and its differences between children of 7, 9, and 12 years of age. Certain tendencies in the temperature distribution patterns could be observed in thermal images. The highest values were observed around the shoulder belt and in the trunk, whereas the lower extremities showed the lowest temperature. Such thermal distribution was also observed in other studies, e.g., by Dębiec-Bąk et al. in football players [34]. The reason for that is the vasculature system and the presence of inner organs. Our research showed a reduction in average temperature values in all subjects, immediately after physical exercises. The highest drop could be observed in the group of 12-year-olds. It may indicate the highest level of development of the body cooling processes in the group of the oldest subjects. Effective sweating mechanisms facilitate the removal of excess heat from the body during exercise. Inbar et al. [35] compared thermoregulatory processes due to physical effort in three age groups: boys, young men, and older men. The analysis of the results showed that the highest level of sweat secretion, which consequently cools down the body during exercise, is observed in young men. The oldest subjects were characterized by low efficiency of the sweating process, whereas the youngest boys had the lowest sweating levels.

### Particularities of thermoregulation in children

The thermoregulation center gains the full independence between the age of 1 and 2 years. This capacity is lost again around the sixth decade of life, which results in similar, somehow impaired, thermoregulatory effectiveness of children and the elderly. Inoue et al. [36] in their research investigated how the ability to lose excess heat changes depending on age. Their results exhibited that children have low capacity to sweat in comparison with adults. Elimination of excess heat occurs by vasodilation of the skin vasculature. However, a high surface area to weight ratio can expose a child to excessive heat absorption in a hot climate. They also observed that due to the processes of involution, thermoregulatory abilities are again reduced in the elderly. Leites et al. [37] conducted an analysis of thermoregulatory processes in men and 10-year-old boys taking into account the body mass in both groups. The subjects performed 80 min of physical exercises in the form of cycling in four 20-min units. The lower production of sweat and heat energy per unit of body surface area in boys were observed. In addition, it was noted that men produced more mechanical energy of muscle work compared to younger subjects. Similar results were obtained by Shibasaki et al. [38], who compared the thermoregulatory response of the body in boys (10–11 years of age) and young men (21–25 years) performing moderate-intensity exercise. The differences between the groups concerned the amount of sweat secretion, which were higher in older subjects. These observations mentioned above and confirmed in our study indicate the existence of a relationship between the degree of body cooling after exercises and age.

### Thermoregulation is efficient adapting human body to physical exercises

An efficient thermoregulation mechanism enables the adaptation of the human body to physical exercises [39]. It is also vital to avoid the negative impact of heat on health and productivity, e.g., during physical work [40]. Cholewka et al. [41] examined by means of thermography the correlation between recorded thermal parameters during endurance effort and the generated power. Strong correlation between these parameters was recorded. This proves the usefulness of thermography as a method for analyzing energy expenditure and also for designing the tests performed during the training cycle.

In the research presented by Duffield et al. [42], it was shown that people undertaking regular physical activity have better efficiency of maintaining thermal balance. Their defense mechanisms operate more efficiently than in non-training people. Regular physical activity affects thermoregulatory processes. This research and our examination may be a hint for parents, demonstrating that a systematic and well-chosen form of training can have a positive impact on the development of their child’s thermoregulatory processes.

### Interpretation of temperature patterns after exercise and recovery time

In our research, after the recovery time (measurement 3), temperature values in children aged 7 and 9 were close to initial ones. However, the group of 12-year-olds was the only one which, after recovery time, did not reach the parameters recorded before exercises. This may be due to the fact that the oldest subjects showed the largest decreases in body temperature after general fitness training. The increase of the body surface temperature after the recovery time has also been demonstrated in studies by Chudecka and Lubkowska [43] using thermal recording. Having analyzed the temperature values in a group of volleyball players after a 90-min workout and a 10-min recovery time, they observed a drop of temperature immediately after physical exercise, while an increase in surface body temperatures followed the recovery time. These researchers conducted an analogous research in a group of handball players. The analysis of surface body temperature after a 10-min recovery time showed an increase in the temperature of the examined areas in all participants. In another study, Chudecka and Lubkowska [44] noted that a return to the initial temperature after a recovery time is more effective in people with a higher value of maximum oxygen uptake. The human body during physical activity has a higher body temperature, due to the increased metabolism of the individual systems. In physiological response to such stimulus as training, cooling mechanisms protecting against hyperthermia are activated, mainly due to increased sweating and vasodilation. Efficient thermoregulation processes are also responsible for returning to pre-exercise temperature. The ability to maintain the thermal balance during daily physical activities and exercises is crucial for the proper functioning of the body [445]. The performed in our group analysis revealed that after physical exercises, a statistically significant decrease in temperature was noted in all age groups, in all areas of the body. The largest temperature reduction was observed in 12-year-olds, while the smallest temperature drop was recorded in the youngest subjects (7-year-olds). After a given recovery time, the temperature did not return to the initial values only in the group of 12-year-olds. The observed significant differences in the body surface temperature between the group of 7-year-olds and 12-year-olds indicate the need to differentiate the level of exercise intensity depending on the age.

When planning training load and physical activity in school curricula, one should take into account individual reactions of thermoregulatory mechanisms in children, as well as the criterion of children’s age. Trainers and teachers of sports activities in schools should be acquainted of the thermoregulatory efficiency of the child’s body what may constitute the basis for prevention against injuries and strain of the locomotory system in school children.

## Concluding remarks and expert recommendations in the framework of 3P medicine

### Thermoregulation is highly individual and predictive for potentially cascading pathologies

Well-controlled thermoregulation is crucial for physical and mental human health. A relatively narrow body temperature range of 36.5–37 °C is rigorously kept allowing for timely enzymatic reactions by the optimal kinetic window and, therefore, an effective performance of all physiological processes. To this end, feeling cold is a normal response towards changing external temperatures, in order to win back the thermal comfort by physical activity and energy supply based on well-concerted regulation mechanisms. At low temperatures, an adaptive vasoconstriction and an increase in blood pressure and heart rates synergistically result in heat production and conservation for maintaining the optimal body temperature [46]. Maintaining thermal comfort is energetically costly [47] and demands well-organized mitochondrial functionality. Mitochondriopathies causing dysfunction of the mitochondrial respiratory chain are characteristic for several pathologic conditions such as cancerous Warburg transformation and neurodegenerative disabilities [48–50].

For example, abnormal thermoregulation has been demonstrated for breast cancer patients, who are frequently deficient in achieving thermal comfort [51]. They feel excessively hot or cold, when disease-free attenders are well comfortable with ambient temperature conditions [46]. To this end, pro-inflammatory cytokines (IL-1, IL-6, TNF-α) regulating the immune reactions [52] are frequently overexpressed by cancer patients [53] affecting thermoregulation by activating the cyclooxygenase 2 and production of prostaglandins [54].

Another example: detectable brain temperature disturbances and brain-systemic temperature decoupling are involved in the stroke pathology [55]. Consequently, an altered brain thermoregulation may serve as a neuroimaging biomarker in CNS injury.

In conclusion, altered and deficient thermoregulation is considered an important diagnostic indicator which can be of great clinical utility for specialized screening programs and individualized prediction of pathologies by individualized patient profiling [56]. Contextually, altered thermoregulation patterns are instrumental for specific phenotypes useful for detection of suboptimal health conditions that can be exemplified by the Flammer syndrome phenotype and associated cascading pathologies [51, 57, 58].

### Body exercise-based disease prevention is effective when adapted individually: multi-parametric guidance for prescribing exercises is needed

As stated above, physiologic versus abnormal thermoregulation is functionally linked with regular physical activities and optimal energy supply. These individual components are well-concerted together via systemic effects being therefore highly indicative for individual healthcare status and prediction of associated pathologies. In this context, individually adapted physical activities are crucial for the targeted disease prevention as stated in both *EPMA Position Paper 2021* [59] and a *joint position paper of the Suboptimal Health Study Consortium and European Association for Predictive, Preventive and Personalised Medicine EPMA J. 2021* [60]. To this end, topic-dedicated research groups emphasize an importance of diversified physical activities in early childhood. Thus, most recently performed studies indicate that diversified physical activity at age six is important for developing optimal physical activities in general and, in particular, motor competence in adolescence. The authors do emphasize the point that not only the amount and intensity but specifically an increased diversity of children’s daily physical activities is decisive for optimal behavioral patterns and health promotion later on in life [61].

Noteworthy, socioeconomic status was demonstrated to be inversely associated with outcomes related to the intensity and quality of youth physical and sports activity. To this end, a cross-sectional survey with 1038 students in 5–12th grades in the USA (King County and Washington including 50% girls, 58% non-White, and 32% from homes where languages other than English are spoken) was conducted. Responders described their physical activity and sports experiences as well as demographic factors such as family affluence categorized as low, medium, and high [62]. For children from low-affluence families, lower intensity of physical activity and rates of ever playing sports were reported. The barriers to sports these children described are “don’t feel welcome on teams” and “too expensive,” among others. The disparity results in three times higher odds of meeting physical activity recommendations as well as three times higher odds of ever participating in sports reported for middle school children from high-affluence families compared to peers from low-affluence families. Consequently, socioeconomical particularities have to be taken into consideration for coaching individually adapted physical activities to reach satisfactory health promoting benefits.

American College of Sports Medicine position stand stated that although general recommendations for physical exercises have been elaborated, *the exercise program should be modified according to an individual’s habitual physical activity, physical function, health status, exercise responses, and stated goals* [63]. Further, *behaviorally based exercise interventions, the use of behavior change strategies, supervision by an experienced fitness instructor, and exercise that is pleasant and enjoyable can improve adoption and adherence to prescribed exercise programs.*

Body temperature measurements are instrumental for personalized couching towards regular physical activities in school children as demonstrated in this article.

## Data Availability

All study relevant data are presented in the manuscript.
